# The effect of serum IL-2 levels on the prognosis of primary biliary cholangitis-related liver failure and the preliminary exploration of its mechanism

**DOI:** 10.3389/fimmu.2022.995223

**Published:** 2022-09-08

**Authors:** Qi Wang, Yang Wang, Wenying Qiao, Bin Xu, Yanmin Liu, Xiaodan Zhang, Wenjuan Li, Juan Zhao, Mengcheng Liu, Yang Zhang, Dexi Chen, Chunyang Huang, Ronghua Jin

**Affiliations:** ^1^ Beijing Institute of Hepatology, Beijing You ‘an Hospital, Capital Medical University, Beijing, China; ^2^ Second Department of Liver Disease Center, Beijing You ‘an Hospital, Capital Medical University, Beijing, China; ^3^ National Center For Infectious Diseases, Beijing Ditan Hospital, Capital Medical University, Beijing, China

**Keywords:** IL-2, primary biliary cholangitis, liver failure, prognosis, immune

## Abstract

**Background:**

In primary biliary cholangitis (PBC), the levels of serum IL-2 were involved in liver inflammation and immune changes. This study aimed to investigate the prognostic significance of serum IL-2 combined with total bilirubin (TBIL) in liver failure and cytokine changes during the disease.

**Methods:**

A total of 160 PBC patients treated with UDCA were included. Parameters at admission were collected, and the COX regression model was used to predict independent risk factors associated with PBC disease progression. We identified the optimal cut-off values and prognosis effects of serum IL-2 and TBIL based on the time-dependent receiver operating characteristic (ROC) curve. We also analyzed the incidence of liver failure with Kaplan-Meier survival analysis. In addition, the changes of cytokines (mainly IL-2) in liver tissues and blood samples from 11 patients with end-stage PBC liver failure and five healthy controls were examined.

**Results:**

Age, IL-2, ALB, γ-GT, ALP, TBIL, Hb, TBA, WBC, and PLT, as well as anti-Sp100, were found to be independent risk factors in PBC patients with liver failure. Patients with decreased serum IL-2 levels and increased TBIL levels have a significantly higher incidence of liver failure and a worse prognosis. Patients with advanced PBC liver failure after liver transplantation exhibited a significant decrease in levels of serum IL-2 and a relatively immunosuppressed status.

**Conclusions:**

The combination of serum IL-2 and TBIL can be a predictor of the progression of liver failure in patients with primary biliary cholangitis, and it is likely to be related to the expression of GM-CSF and G-CSF.

## Introduction

Primary biliary cholangitis (PBC) is a specific immunoreactive liver disease characterized by positive anti-mitochondrial antibodies (AMA). In recent years, the incidence of PBC in the Asia-Pacific region has gradually increased, with the prevalence rate reaching 118.75 cases per million (1). The prognosis of PBC has always been a hot issue of clinical concern (2). Total bilirubin (TBIL) levels were used to predict prognosis from an early stage in PBC patients. However, due to poor prognosis of PBC patients, TBIL lacks early warning and intervention values (3).

Multiple elements influence the occurrence and development of PBC. In the early stages of the disease, various factors, including genes and environment, disrupt liver immune tolerance, resulting in abnormal immune system activation in patients with PBC (4), which further cause the damage of intrahepatic bile duct epithelial cells (BECs). When a number of exogenous antigens (such as bacteria, etc.) are absorbed through the portal vein circulation and contact the small bile ducts, BEC actively binds to external antigens through its surface receptors, then secreting cytokines such as IL-2 (5) to promote its apoptosis. In the process of BEC apoptosis, the E2 component of the pyruvate dehydrogenase complex(PDC-E2) antigen epitope is exposed, triggering immune cells to attack adjacent BECs one after another (6), causing continuous damage and disappearance of the small bile ducts, which in turn leads to liver fibrosis (7, 8).

As the disease progresses, cytokine levels consequently change. Studies have confirmed that the level of Th1 cells in peripheral blood was higher in patients with early-stage PBC than in late-stage when staging is performed by pathological liver biopsy. Advanced intrahepatic Th1 and its secretion of IL-2 levels were low (9–11), which promotes the development of the liver toward fibrosis. The level of IL-2 may be related to the prognosis of patients with PBC. Therefore, by collecting clinical indicators and cytokine detection in patients with PBC, this study explores the change of IL-2 levels in patients with PBC during liver failure and analyzes whether IL-2 in combination with TBIL improves the predictive capacity. In addition, we examined cytokines in patients with PBC liver failure after liver transplantation to observe the changes in cytokine levels (mainly IL-2) in the early stages of PBC onset and end-stage liver failure.

## Materials and methods

### Study subjects

The present study enrolled 160 PBC patients treated with UDCA at Beijing You ‘an Hospital affiliated with Capital Medical University from January 1, 2010, to July 16, 2018. And Qingdao Liver Transplant Institute (Remaining specimens trimmed from a liver transplant donor) provided donor and preoperative serum of 11 PBC liver transplant patients and five healthy controls.

The diagnosis of PBC was referred to the 2017 guidelines for PBC diagnosis issued by the European Society of Hepatology (12) and the American Association for Research on Liver Disease (13) and met any two or three of the following criteria: (i) Biochemical evidence of cholestasis, mainly characterized by elevated serum alkaline phosphatase (ALP) levels, excluding extrahepatic biliary obstruction; (ii) serum antimitochondrial antibody (AMA) positive; (iii) liver wear histological examination showing nonsuppurative destructive cholangitis with interlobular bile duct destruction. Exclusion criteria included (i) incorporation of autoimmune hepatitis or extrahepatic autoimmune diseases; (ii) infection with hepatitis A, B, C, D, and E; EB virus, cytomegalovirus, or human immunodeficiency virus; (iii) incorporation of other liver diseases such as alcoholic liver disease, drug liver injury, and (iv) the utilize of corticosteroids or immunosuppressive drugs for more than two weeks. Hepatic failure in this study was defined as coagulation dysfunction [prothrombin activity (PTA) <40% or International Standardized Ratio (INR) ≥1.5] and jaundice [total serum bilirubin (TBIL) ≥171μmol/L or daily elevation≥17.1μmol/L].

The study was conducted according to the 1975 Declaration of Helsinki. All the participating institutional research committees approved the protocol under local regulations.

### Data collection

The data from 160 patients were collected at the time of initial patient admission. Mainly included demographic data [age, sex], Multi-cytokine detection [interleukin-10 (IL-10), interleukin-2 (IL-2), interleukin-4 (IL-4), interleukin-6 (IL-6), interferon-γ(INF-γ), granulocyte-macrophage colony-stimulating factor (GM-CSF)], laboratory examination indicators (alanine transaminase (ALT), aspartate transaminase (AST), γ-glutamyl transpeptidase (γ-GT) and alkaline phosphatase (ALP), total bile acid (ALB), total bilirubin (TBIL), albumin (ALB), International standardized ratio (INR), immunoglobulin triple [immunoglobulin A (IgA), immunoglobulin (IgG), immunoglobulin G, immunoglobulin M (IgM), and associated autoantibody examination [anti-nuclear antibody (ANA), antimitochondrial antibody (AMA), anti-Sp100 antibody, anti-gp210 antibody].et.

In addition, we collected serum and liver tissue specimens from 11 patients with liver failure and five healthy controls and stored them in a refrigerator at -80°C.

### Cytokine assay

100mg of liver tissue was ground and lysed in Lysis Buffer on ice for 30min. After a 30-minute-centrifuge at 14000g, the supernatant was collected. The tissue lysate supernatant and serum samples were subjected to cytokine detection using the Human Cytokine/Chemokine Panel I (Merck, HCYTA-60K) kit, and the MILLIPLEX liquid phase mass spectrometry was used for detection.

### Statistical analysis

All data were analyzed with SPSS26.0 software (IBM Corporation, Armonk, NY, USA). Continuous variables were expressed as mean ± standard deviation (SD), and an independent sample t-test was used to compare groups. Categorical variables were described in frequency and percentage, and the chi-square test compared groups. Univariate and multivariate Cox regression analysis models were performed to identify independent risk factors associated with disease progression. The Kaplan-Meier method was applied to observe the progression of the disease. The cut-off value was determined based on the ROC curve and the Youden index. P<0.05 was considered statistically significant.

## Results

### Baseline characteristics and laboratory examination of PBC patients

Of the 160 patients with PBC, 23 (14.4%) were men, and 137 (85.6%) were women. The mean age was 51 years (20 – 84 years) and the median follow-up time was 56 months (1 – 140 months) (**Table 1**).

**Table 1:** Baseline information and laboratory parameters of PBC patients.

Variables	Mean ± SD/n (%) (n=160)
Age (years old)	51.35 ± 11.47
Gender, male/female (%)	23(14.4%)/137(85.6%)
IL-10 (p g/ml)	4.96 ± 7.75
IL-2 (p g/ml)	7.47 ± 10.29
IL-4(p g/ml)	7.21 ± 24.50
IL-6(p g/ml)	31.35 ± 80.21
IFN-γ (p g/ml)	26.67 ± 97.50
GM-CSF (p g/ml)	10.48 ± 34.01
ALB(g/dL)	37.90 ± 6.85
γ-GT(IU/L)	268.52 ± 331.02
ALP(IU/L)	238.39 ± 222.321
TBA(μmol/L)	71.28 ± 80.28
TBIL(μmol/L)	64.42 ± 82.93
CHOL (mg/dL)	5.29 ± 4.37
ALT(IU/L)	201.11 ± 485.95
AST(IU/L)	212.35 ± 468.75
IgG(mg/dL)	18.09 ± 6.78
IgA(mg/dL)	3.59 ± 1.78
IgM(mg/dL)	3.22 ± 2.43
anti-Sp100, positive/negative (%)	22(13.8%)/138(86.2%)
anti-Gp210, positive/negative (%)	42(26.3%)/118(73.7%)
ANA, positive/negative (%)	142(88.8%)/18(11.2%)

IL-10, interleukin-10; IL-2, interleukin-2; IL-4, interleukin-4; IL-6, interleukin-6; IFN-γ, interferon-γ; GM-CSF, Granulocyte-macrophage colony-stimulating factor; ALB, albumin; γ-GT, γ-glutamyl transpeptidase; ALP, alkaline phosphatase; TBA, total bile acid; TBIL, total bilirubin; CHOL, cholesterol total; ALT, alanine aminotransferase; AST, aspartate aminotransferase; IgG, immunoglobulin G; IgM, immunoglobulin M; IgA immunoglobulin A; ANA, antinuclear antibodies.;anti-Sp100, anti-Sp100 antibody; anti-Gp210, anti-Gp210 antibody.

As of January 1, 2021, 18 patients (11.3%) have developed liver failure (**Table 2**). The levels of cytokines, such as IL-10, IL-2, IL-4, IL-6, IFN-γ, and GM-CSF in PBC patients who developed liver failure, were significantly reduced, and the difference in IL-2 between the two groups was statistically significant (P=0.028). Among the liver function biochemical indicators, the levels of ALT, AST, TBA, and TBIL were increased, and the difference in TBA between the two groups was statistically considerable (P <0.05). For autoantibody detection, anti-Sp100, anti-gp210, and ANA were statistically meaningful between the two groups (P <0.01).

**Table 2:** Comparison of baseline clinical and laboratory parameters levels at entry between patients with and without liver failure.

Parameters	Patients not developing liver failure (n=142)	Patients who developed liver failure (n=18)	P-value
Age (years old)	50.72 ± 11.44	56.35 ± 10.80	**0.050**
Gender, male/female (%)	20(14.1%)/122(85.9%)	3(16.7%)/15(83.3%)	**0.000**
IL-10(p g/ml)	5.13 ± 8.11	3.69 ± 3.83	0.430
IL-2(p g/ml)	8.11 ± 10.75	2.46 ± 1.93	**0.028**
IL-4(p g/ml)	7.73 ± 25.93	3.18 ± 4.31	0.459
IL-6(p g/ml)	33.58 ± 84.70	15.37 ± 19.03	0.366
IFN-γ (p g/ml)	26.97 ± 102.73	24.26 ± 37.26	0.912
GM-CSF (p g/ml)	10.67 ± 35.87	9.01 ± 12.18	0.846
ALB(g/dL)	38.13 ± 7.03	36.07 ± 5.08	0.231
γ-GT(IU/L)	264.69 ± 332.40	298.33 ± 327.86	0.686
ALP(IU/L)	239.30 ± 225.44	231.33 ± 202.17	0.887
TBA(μ mol/L)	64.62 ± 73.01	126.08 ± 113.79	**0.044**
TBIL (μ mol/L)	59.10 ± 78.67	106.39 ± 104.39	0.079
CHOL (mg/dL)	5.22 ± 4.27	5.89 ± 5.25	0.553
ALT(IU/L)	181.77 ± 467.62	351.52 ± 604.88	0.265
AST(IU/L)	193.52 ± 462.57	358.79 ± 504.07	0.201
IgG(mg/dL)	18.14 ± 6.93	17.69 ± 4.98	0.792
IgA(mg/dL)	3.55 ± 1.76	3.85 ± 1.94	0.503
IgM(mg/dL)	3.20 ± 2.44	3.33 ± 2.42	0.842
anti-Sp100, positive/negative (%)	18(13.8%)/124(86.2%)	4(22.2%)/14(77.8%)	**0.000**
anti-gp210, positive/negative (%)	37(26.1%)/105(73.9%)	5(27.8%)/13(72.2)	**0.000**
ANA, positive/negative (%)	127(89.4%)/15(10.6)	15(83.3%)/3(16.7%)	**0.000**

IL-10, interleukin-10; IL-2, interleukin-2; IL-4, interleukin-4; IL-6, interleukin-6; IFN-γ, interferon-γ; GM-CSF, Granulocyte-macrophage colony-stimulating factor; ALB, albumin; γ-GT, γ-glutamyl transpeptidase; ALP, alkaline phosphatase; TBA, total bile acid; TBIL, total bilirubin; CHOL, cholesterol total; ALT, alanine aminotransferase; AST, aspartate aminotransferase; IgG, immunoglobulin G; IgM, immunoglobulin M; IgA immunoglobulin A; ANA, antinuclear antibodies.;anti-Sp100, anti-Sp100 antibody; anti-gp210, anti-gp210 antibody. All bold letters represent P values less than 0.05 and are statistically significant.

### Prognostic factors associated with liver failure

Univariate analysis revealed that the incidence of liver failure was remarkably associated with age, IL-2, γ-GT, TBIL, WBC, and anti-Sp100. Moreover, multivariate analysis showed that age(P=0.023), IL-2(P=0.023), TBIL(P=0.001), and anti-SP100(P=0.001) were independent predictors of the development of liver failure (**Table 3**).

**Table 3:** Cox regression analysis of risk factors for liver failure in primary biliary cirrhosis.

variables	Univariate		Multivariate	
	HR (95%)	P-value	HR (95%)	P-value
Age (years old)	1.065 (1.012-1.121)	**0.015**	1.062 (1.008-1.118)	**0.023**
IL-2 (p g/ml)	0.771 (0.606-0.981)	**0.034**	0.779 (0.628-0.966)	**0.023**
IFN-γ (p g/ml)	1.003 (0.996-1.010)	0.386		
GM-CSF (p g/ml)	0.970 (0.931-1.011)	0.145		
γ-GT (IU/L)	1.002 (1.000-1.004)	**0.031**		
ALP (IU/L)	0.997 (0.993-1.001)	0.148		
TBIL (μ mol/L)	1.008 (1.003-1.013)	**0.001**	1.008 (1.003-1.012)	**0.001**
Hb (g/L)	0.981 (0.962-1.001)	0.068		
WBC (×10^9/L)	0.669 (0.472-0.946)	**0.021**		
anti-SP100 (%)	2.285 (1.448-3.606)	**0.000**	1.945 (1.313-2.881)	**0.001**
anti-Gp210 (%)	1.400 (0.920-2.430)	0.117		
ANA (%)	0.800 (0.496-1.289)	0.359		

IL-2, interleukin-2; IFN-γ, interferon-γ; GM-CSF, Granulocyte-macrophage colony-stimulating factor; γ-GT, γ-glutamyl transpeptidase; ALP, alkaline phosphatase; TBIL, total bilirubin; HB, hemoglobin; anti-Sp100, Anti-Sp100 antibody; anti-Gp210, anti-Gp210 antibody; ANA, antinuclear antibodies. All bold letters represent P values less than 0.05 and are statistically significant.

### Correlation of the serum IL-2 and the clinical indicators

**Figure 1:**
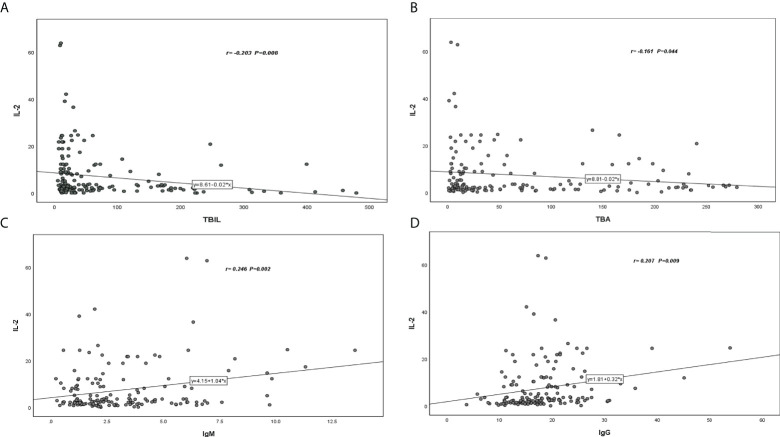
Correlation coefficient and P -value between serum IL-2 level and various parameters of patients with PBC. **(A)** Serum IL-2 was negatively correlated with TBIL (r=-203). **(B)** Serum IL-2 was negatively correlated with TBA (r=-161). **(C)** Serum IL-2 was positively correlated with IgM (r=0.246). **(D)** Serum IL-2 was positively correlated with IgG(r=0.207).

Serum IL-2 was negatively correlated with TBIL (r=-203) and TBA (r=-161) (**Figures 1A, B**), while IgM (r=0.246) and IgG (r=0.207) showed a positive correlation with serum IL-2(**Figures 1C, D**).

### Serum IL-2 in combination with TBIL

**Figure 2:**
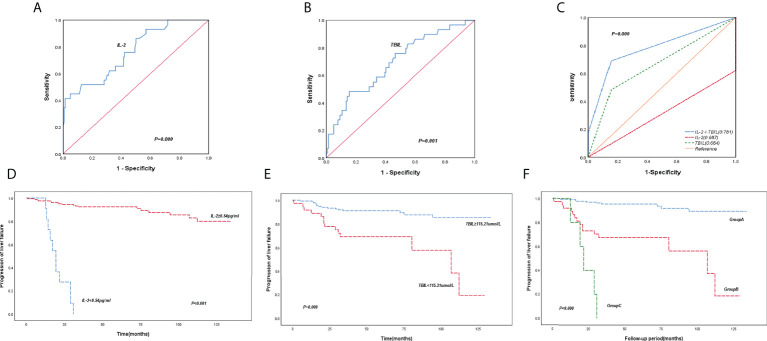
ROC curve and Kaplan-Meier survival curve of IL-2 combined with TBIL. **(A)** ROC curves for IL-2. **(B)** ROC curves for TBIL. **(C)** ROC curve of IL-2 combined with TBIL. **(D, E)** Cumulative survival of PBC patients were analyzed by the Kaplan-Meier method, using the baseline IL-2 and TBIL levels. **(F)** Subgroup studies were stratified for the incidence of liver failure in patients by the Kaplan-Meier method based on the levels of baseline IL-2 and TBIL.

Based on the time-dependent ROC curves and Youden’s index, the cut-off values for the serum IL-2 level and TBIL were 0.54pg/ml and 115.21μmol/L, respectively. The time-dependent ROC curves and areas under the curves (AUCs) showed that (**Figure 2C**) the level of IL-2 combined TBIL (AUC=0.781, P=0.000) was superior to IL-2 (AUC=0.687, P=0.000) (**Figure 2A**) and TBIL levels alone (AUC=0.664, P=0.001) (**Figure 2B**). According to Kaplan-Meier analysis (**Figures 2D, E**), the incidence of liver failure was markedly increased when IL-2 levels were ≤ below 0.54 pg/ml or TBIL ≥ 115.21 μmol/ml. Conversely, when IL-2 level ≥ 0.54pg/ml or TBIL <115.21μmol/ml, the incidence of liver failure in patients decreased dramatically.

Subgroup analysis of the incidence of liver failure based on the cut-off values of IL-2 and TBIL was divided into three groups (**Figure 2F**). Group A (IL-2≥0.54 pg/ml and TBIL ≤ 115.21μmol/ml), Group B (IL-2≥0.54 pg/ml and TBIL>115.21umol/L or IL-2 <0.54 pg/ml and TBIL ≤ 115.21μmol/ml), and Group C (IL-2 <0.54 pg/ml and TBIL≥115.21μmol/ml). The Kaplan-Meier analysis indicated a slowly escalating prevalence of liver failure in Group A patients, while the rate was substantially higher in group C patients. The difference between the groups was notable (P <0.001).

### IL-2 depletion in peripheral blood due to recruitment of IL-2 by GM-CSF in the liver and altered immune status in the patient’s blood

**Figure 3:**
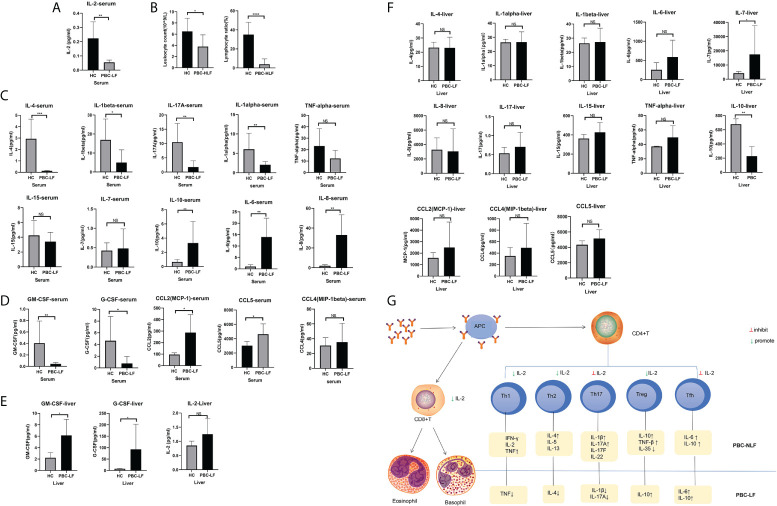
Cytokine changes in patients transplanted with advanced PBC liver failure after liver transplantation. **(A)** IL-2 levels in patients with advanced PBC liver failure after liver transplantation. **(B)** Changes in the proportion of leukocytes and lymphocytes in liver transplant patients compared to healthy controls **(C)** Treatment of cytokine changes in liver transplant patients’ blood compared to healthy controls **(D)** Changes in colony-stimulating factors and chemokines in liver transplant patients’ blood compared to healthy controls **(E)** Expression of IL-2, GM-CSF, and G-CSF in liver transplant patients’ liver tissues compared to healthy controls **(F)** Changes in various cytokines in liver transplant patients’ liver tissue compared to healthy controls. **(G)** PBC immune cell type changes. *P < 0.05; **P < 0.01; ***P < 0.001. NS is the abbreviation of Normal Saline, which stands for the control group.

To verify the above results and explore the primary mechanism, we further detected the serum cytokine levels of PBC liver failure after liver transplantation. The outcomes demonstrated that serum IL-2 levels in patients with advanced PBC liver failure after liver transplantation were lower than in healthy controls (**Figure 3A**). In addition, we performed blood cell count statistics and observed that patients with PBC-LF had lower leukocytes and reduced lymphocyte rates than healthy controls (**Figure 3B**). Furthermore, several common T lymphocytes secreted cytokines were further tested, showing that, except for a slight increase in serum IL-6 levels and an obvious increment in the acute pro-inflammatory factor IL-8, a large number of classic pro-inflammatory cytokines showed a state of being equal to or even decreasing compared to the control. For example, the serum levels of IL-4, IL-1β, and IL-17A decreased, and the serum levels of pro-inflammatory cytokines TNF-α, IL-15, and IL-17 were close to healthy controls. Moreover, the classic anti-inflammatory cytokine IL-10 increased visibly (**Figure 3C**), which was inconsistent with the high immune state in PBC patients, presenting a relatively immunosuppressive state.

To further validate this phenomenon, we also examined other cytokines in which the colony-stimulating factors GM-CSF and G-CSF were lower than normal control levels, chemokine CCL2 and CCL5 were higher in the peripheral blood than in healthy controls, and CCL4 showed an upward trend but insignificant (**Figure 3D**). Additionally, the pro-inflammatory cytokine IL-1alpha was noticeably diminished and IL-15 and IL-17 converged to normal levels compared to healthy controls. We further examined liver cytokine levels in PBC liver patients using the remaining donor tissue as a control to support this conclusion. The results revealed that the majority of the cytokines in the tissue are close to normal and healthy controls (**Figure 3F**). Among them, GM-CSF and G-CSF were increased, and IL-2 was marginally raised (**Figure 3E**).

In conclusion, patients with PBC liver failure might have a relatively immunosuppressed state (**Figure 3G**), predominantly caused by lymphocytopenia, which was probably related to the intervention of IL-2 on CD4 cell differentiation. This phenomenon was likely influenced by GM- CSF, and G-CSF.

## Discussion

PBC, formerly known as primary biliary cirrhosis, is an autoimmune disease characterized by progressive damage to the small intrahepatic bile ducts. If left untreated, it can eventually progress to cirrhosis or even liver failure (14, 15). Currently, UDCA is the first-line therapeutic drug for patients with PBC (16). However, due to limited treatment methods, the disease often progresses rapidly (17). Therefore, it is critical to identify the factors that affect the patient’s overall condition to further improve the prognosis.

The level of serum TBIL principally, reflecting the degree of bile duct destruction and cholestasis in PBC patients, is a powerful predictor of clinical endpoint events, having great values for the prognostic assessment (18). Under normal circumstances, the TBIL of patients was unchanged but increased to varying degrees as the disease progresses. Moreover, it has been reported that serum bilirubin levels and Mayo risk score have good value for determining PBC prognosis (19). The Mayo risk score majorly includes bilirubin, albumin, age, prothrombin time, and edema degree (20), where both elevated total bilirubin and prolonged prothrombin time are linked to liver failure. In our study, we demonstrated that TBIL correlated with prognostic survival in patients with PBC, which was consistent with the previous reports. Despite the availability of appropriate treatment, the condition of the patient will still advance very rapidly, when the disease reaches the stage of liver failure.

The pathogenesis of PBC may be related to abnormal immune regulation (21). In the early stage of PBC, there are many Th1 cells infiltrated in the liver tissues of patients (22, 23). The effectors secreted by Th1 cells can antagonize or promote each other, forming a complex network of cytokines to regulate the immune response. IL-2 is a potent mitogen and growth regulator for T cells. After binding to the corresponding receptors on the surface of T lymphocytes, IL-2 can immediately activate JAK1 and JAK3 coupled to the receptors (24, 25), and then promptly cause the tyrosine phosphorylation of STAT3 and STAT5. The over-activated STAT3 and STAT5 could initiate a series of downstream effects (26). To some extent, the level of IL-2 reflects the state of cellular immunity of the body and plays a crucial role in the pathogenesis of various immune diseases (27). Malek constructed a mouse model with IL-2R and concluded that normal development of Treg in the thymus and regular expression of CD25 and Foxp3 are inseparable from IL-2R signaling that could help to maintain CD4 ^+^ CD25 ^+^ CD 25 ^+^ Foxp3 ^+^ Treg in the periphery. And the loss of IL-2R signaling can cause autoimmune diseases (28).In our study, we found that IL-2 is an independent risk factor for disease progression(P<0.005), patients with decreased serum IL-2 levels and increased TBIL levels have a remarkable higher incidence of liver failure and poorer prognosis, which was verified by the ROC curve of IL-2 combined with TBIL, with an AUC of 0.781.

Recent studies have taken IL-2’s signaling pathways that control the differentiation and homeostasis of pro-inflammatory and anti-inflammatory T cells as the basis of immunomodulatory molecules, which is a crucial regulator of T cell metabolism (29). Moreover, IL-2 is essential for the development of Tregs in the thymus and the regulation and proliferation of Tregs in peripheral tissues, especially for the transcription program required to maintain Treg function. Our experiment found that the reduction of serum IL-2 levels in patients with advanced PBC liver failure will lead to a decrease in Th1 type cells (serum TNF-α level in the blood approaches the normal value). We also observed a decrease in Th2 cells (lower serum IL-4 levels compared to the control group), an inconsequential increase in Th17 cells (decreased serum IL-17A level and reduced serum IL-1β level as compared to the control group), and an accumulation of Treg cells (marked rise in serum IL-10).

Granulocyte-macrophage colony-stimulating factor (GM-CSF) and IL-2 are the most potent cytokines for the induction of tumor-specific systemic immune responses. GM-CSF performs multiple immunomodulatory activities, including promoting the differentiation of granulocytes, macrophages, and eosinophil precursor cells, stimulating and recruiting DCs (30, 31), and enhancing the expression of IL-2 receptors on the T cell surface (32). The present study suggested that using the fusion protein IL2-GMCSF to augment the levels of IL-2 and GM-CSF remarkably facilitates the cellular activity of DCs, involving phagocytosis, proliferation, and cytokine secretion (33). Another study revealed that GM-CSF is a gateway for lymphocytes and inflammatory cells to invade tissues. By binding to heterodimeric receptors, GM-CSF activates JAK2/STAT5 and Ras/Raf/MAPK pathways and initiates downstream reactions, which could activate macrophages, causing a lot of tissue damage (31). In this study, with massive hepatocyte damage in the advanced stage of PBC, the serum IL-2, GM-CSF, and G-CSF levels were relatively increased and macrophages were activated, with massive hepatocyte damage. These changes may be attributed to the regulation of T cell surface receptors by GM-CSF and G-CSF affecting serum IL-2 levels. The different mechanism has vital research value.

IL-2 is a potent mitogen and growth regulator of T cells, whose expression could determine the composition of CD4(+) T cells and has a decisive influence on the homeostasis and development of T cell subsets (including Th1, Th2, Th17, Tfh). IL-2 can promote the differentiation of CD4(+) T cells into Th1 and Th2 cells and inhibit the differentiation of Th17 and Tfh cells, thus making IL-2 an essential regulator of T cell lineage commitment. In addition, IL-2 signaling pathways, which control the differentiation and homeostasis of pro- and anti-inflammatory T cells, are the basis of immunomodulatory molecules. Therefore, signaling stimulation by IL-2 is essential for the maintenance of regulatory T (T-reg) cells and CD4(+) T cells, as well as for the differentiation of CD8(+) T cells into effector T cells after activation (1). Also, it is crucial for the development of Tregs in the thymus and the regulation, proliferation, and maintenance of Tregs in peripheral tissues. These findings are congruent with our experimental results which found that the reduction of IL-2 in patients with PBC liver failure will lead to the decrease of Th1 type cells (TNF-alpha in the blood is close to the normal value), the Th2 type cells (the decrease of IL-4 in blood compared with the normal control), and the Th17 type cells. The increase of cells was not obvious (the IL-17A in the blood was lower than normal, and the IL-1beta was decreased), and the Treg cells were increased (the IL-10 in the blood was significantly increased).

In our study, 18 patients developed liver failure and all of them exhibited a trend of elevated TBIL. We demonstrated that the combination of IL-2 and TBIL could better predict the outcome of PBC. By examining the correlation of IL-2 with clinical data, we found that reduced IL-2 levels may reflect poor liver function. This study also provides the first evidence for the predictive efficacy of serum IL-2 combined with TBIL in PBC patients with liver failure.

Meanwhile, IL-2 in combination with TBIL can predict the outcome of PBC patients treated with UDCA, which may be related to the expression of GM-CSF and G-CSF. And the fusion protein IL2-GMCSF has been studied to enhance IL-2 and GM-CSF levels to interfere with tumor immune response. This study hopes to provide a new way of predicting the disease development process in PBC patients through our exploration. Moreover, further validation is expected to explore a mechanism to achieve molecular intervention for the prognosis of PBC patients.

There are also some limitations of our study. First of all, this was a single-center retrospective study, which needs to be further verified in multiple centers. Secondly, the clinical sample size of this study is relatively small, and the conclusions need to be validated in a larger range of sample sizes. Then, plasma cytokine levels were tested only once and could not be measured a second time later, making it impossible to assess changes in plasma cytokine levels based on specific outcomes (e.g., improvement/worsening of liver failure). In addition, according to available data, this study determined that serum IL-2 could regulate the immune mechanism of hepatocytes but lacked the exploration of immune mechanism which would be verified in our future experiments by Vivo and Vitro experiments, such as cytokine detection, flow immune cell population analysis and so on.

## Conclusions

Our study, for the first time, demonstrated the prognostic significance of serum IL-2 combined with TBIL and is likely to be related to the expression of GM-CSF and G-CSF in liver failure in PBC patients.

## Data availability statement

The raw data supporting the conclusions of this article will be made available by the authors, without undue reservation.

## Ethics statement

The studies involving human participants were reviewed and approved by the Ethics Committee of Capital Medical University affiliated Beijing You’an Hospital. Written informed consent for participation was not required for this study in accordance with the national legislation and the institutional requirements.

## Author contributions

Conceived and designed the protocol: CH and DC. Collected data: BX and YW. Analyzed data: QW, XZ, and YZ. Statistical analysis: QW. Wrote the manuscript: QW, YW, and WQ. Critically revised and approved the final version of manuscript: RJ and ML. Experiment: YW and QW. Get research funding: WL, JZ, and YL. All authors read and approved the final manuscript.

## Funding

This study was funded by a grant from the Beijing Municipal Administration of Hospitals Incubating Program (Code: PX2019062), 2021 Young and middle-aged Talents Incubation Project (Youth Innovation) of Beijing You ‘an Hospital, Capital Medical University (YNKTQN2021004), Beijing Municipal Institute of Public Medical Research Development and Reform Pilot Project (2021-10), WBE Liver Foundation, (Grant No. WBE2022018), 2020 Science and technology innovation competition of Beijing You ‘an Hospital, Capital Medical University (YAKJCXDS202003)

## Acknowledgments

The authors highly appreciate all patients who participated in the study.

## Conflict of interest

The authors declare that the research was conducted in the absence of any commercial or financial relationships that could be construed as a potential conflict of interest.

## Publisher’s note

All claims expressed in this article are solely those of the authors and do not necessarily represent those of their affiliated organizations, or those of the publisher, the editors and the reviewers. Any product that may be evaluated in this article, or claim that may be made by its manufacturer, is not guaranteed or endorsed by the publisher.
